# *rac*-Ethyl 2-amino-3-hy­droxy-3-[4-(methyl­sulfon­yl)phen­yl]propano­ate

**DOI:** 10.1107/S1600536810052827

**Published:** 2011-01-08

**Authors:** Hao Hu, Yue-Hu Chen, Shao-Song Qian, Shou-Kai Kang

**Affiliations:** a School of Life Science, ShanDong University of Technology, ZiBo 255049, People’s Republic of China

## Abstract

In the title compound, C_12_H_17_NO_5_S, the orientations of the 2-ethyl-2-amino-3-hy­droxy­propano­ate group and the 4-methyl­sulfonyl moiety towards the aromatic ring are periplanar and (−)-anti­clinal, respectively. In the crystal packing, the dominant inter­action is O—H⋯N hydrogen bonding, which generates a chain running along [100]. N—H⋯O and C—H⋯O interactions are also observed.

## Related literature

The title compound is an inter­mediate in the synthesis of florfenicol, a broad spectrum anti­biotic currently used in veterinary medicine, see: Gregory (1957 ▶); Syriopoulou & Harding (1981 ▶).
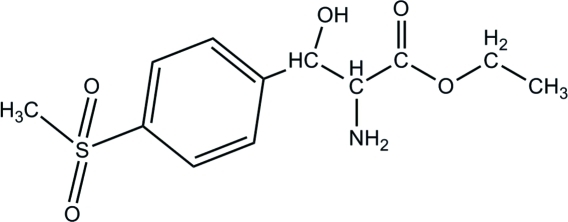


## Experimental

### 

#### Crystal data

C_12_H_17_NO_5_S
*M* *_r_* = 287.33
Triclinic, 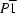

*a* = 4.8123 (11) Å
*b* = 11.382 (3) Å
*c* = 12.637 (3) Å
α = 94.952 (4)°
β = 100.530 (4)°
γ = 94.298 (4)°
*V* = 675.1 (3) Å^3^
*Z* = 2
Mo *K*α radiation
μ = 0.26 mm^−1^
*T* = 273 K
0.18 × 0.16 × 0.10 mm

#### Data collection

Bruker SMART CCD area-detector diffractometer
3543 measured reflections
2363 independent reflections
1786 reflections with *I* > 2σ(*I*)
*R*_int_ = 0.017

#### Refinement

*R*[*F* ^2^ > 2σ(*F* ^2^)] = 0.060
*wR*(*F* ^2^) = 0.179
*S* = 1.04
2363 reflections
174 parameters
H-atom parameters constrained
Δρ_max_ = 0.75 e Å^−3^
Δρ_min_ = −0.23 e Å^−3^

### 

Data collection: *SMART* (Bruker, 2007 ▶); cell refinement: *SAINT* (Bruker, 2007 ▶); data reduction: *SAINT*; program(s) used to solve structure: *SHELXS97* (Sheldrick, 2008 ▶); program(s) used to refine structure: *SHELXL97* (Sheldrick, 2008 ▶); molecular graphics: *PLATON* (Spek, 2009 ▶); software used to prepare material for publication: *SHELXL97*.

## Supplementary Material

Crystal structure: contains datablocks global, I. DOI: 10.1107/S1600536810052827/kp2292sup1.cif

Structure factors: contains datablocks I. DOI: 10.1107/S1600536810052827/kp2292Isup2.hkl

Additional supplementary materials:  crystallographic information; 3D view; checkCIF report

## Figures and Tables

**Table 1:** Hydrogen-bond geometry (Å, °)

*D*—H⋯*A*	*D*—H	H⋯*A*	*D*⋯*A*	*D*—H⋯*A*
O3—H3⋯N1^i^	0.82	1.97	2.784 (3)	174
N1—H1*A*⋯O3^ii^	0.89	2.25	3.088 (3)	158
C7—H7*A*⋯O2^i^	0.96	2.57	3.227 (4)	126

Symmetry codes: (i) 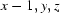; (ii) 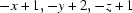.

**Fig. 1.**
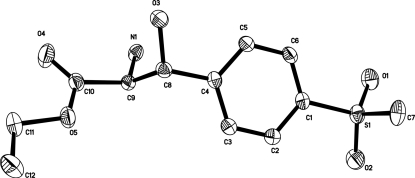
The structure of I showing the atom labelling scheme. Displacement ellipsoids are drawn at the 35% probability level.

**Fig. 2.**
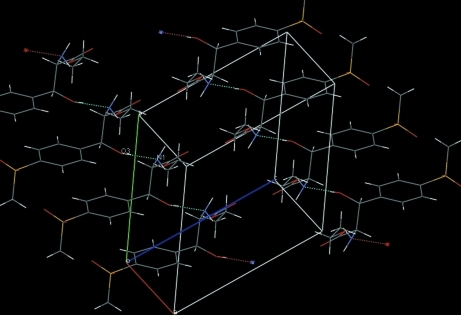
Crystal packing with intermolecular hydrogen bonds generating a chain along [100].

## supplementary crystallographic information

### Comment

The title compound (Fig. 1)is an intermediate of florfenicol, a fluorinated synthetic analog of thiamphenicol, which is currently indicated for the treatment of bovine respiratory disease (BRD) associated with Mannheimia (Pasteurella) haemolytica, Pasteurella multocida, and Haemophilus somnus, and for treatment of bovine interdigital phlegmon (foot rot, acute interdigital necrobacillosis, infectious pododermatitis) associated with Fusobacterium necrophorum and Bacteroides melaninogenicus (Syriopoulou, *et al.*, 1981). The chiral title molecule crystallises in centrosymmetric triclinic space group implying a racemic crystal. The two stereogenic centres C8 and C9 are of opposite chirality. The dominant interaction is hydrogen bond O3-H···N1 which generates a chain along the direction [100] (Fig. 2).

### Experimental

The compound (rac)-ethyl 2-amino-3-hydroxy-3-(4-(methylsulfonyl)phenyl) propanoate was synthesised according to a US patent (Gregory, 1957). 4-(Methylsulfonyl)benzaldehyde (18.4 g, 0.1 mol) reacted with 2-aminoacetic acid (7.5 g, 0.1 mol) and potassium carbonate (29.2 g, 0.21 mol). As a result, 14.2 g of (rac)-2-amino-3-hydroxy-3-(4-(methylsulfonyl)phenyl)propanoic acid was obtained (yield, 55%). The amount of 9.3 g of the title compound was obtained through the reaction of (rac)-2-amino-3-hydroxy-3-(4-(methylsulfonyl)phenyl)propanoic acid (14.2 g, 0.055 mol), ethanol (3.3 mL) and 20 mL of sulfuric acid under reflux for 30 min. Subsequently, 0.1 g of the title compound was dissolved in tetrahydrofurane (5 mL) and single crystals suitable for X-ray diffraction were obtained by spontaneous evaporation of the solution.

### Refinement

All the H atoms attached to C atoms were placed in geometrical positions and constrained to ride on their parent atoms with C—H distance in the range 0.93–0.98 Å, They were treated as riding atoms, with *U*_iso_(H) = 1.2*U*_eq_(C).

### Crystal data

**Table:**

C_12_H_17_NO_5_S	*Z* = 2
*M**_r_* = 287.33	*F*(000) = 304
Triclinic, *P*1	*D*_x_ = 1.414 Mg m^−^^3^
Hall symbol: -P 1	Mo *K*α radiation, λ = 0.71073 Å
*a* = 4.8123 (11) Å	Cell parameters from 1152 reflections
*b* = 11.382 (3) Å	θ = 2.3–25.9°
*c* = 12.637 (3) Å	µ = 0.26 mm^−^^1^
α = 94.952 (4)°	*T* = 273 K
β = 100.530 (4)°	Prism, colourless
γ = 94.298 (4)°	0.18 × 0.16 × 0.10 mm
*V* = 675.1 (3) Å^3^	

### Data collection

**Table:**

Bruker SMART CCD area-detector diffractometer	1786 reflections with *I* > 2σ(*I*)
Radiation source: fine-focus sealed tube	*R*_int_ = 0.017
graphite	θ_max_ = 25.1°, θ_min_ = 1.7°
φ and ω scans	*h* = −4→5
3543 measured reflections	*k* = −13→13
2363 independent reflections	*l* = −15→9

### Refinement

**Table:**

Refinement on *F*^2^	Primary atom site location: structure-invariant direct methods
Least-squares matrix: full	Secondary atom site location: difference Fourier map
*R*[*F*^2^ > 2σ(*F*^2^)] = 0.060	Hydrogen site location: inferred from neighbouring sites
*wR*(*F*^2^) = 0.179	H-atom parameters constrained
*S* = 1.04	*w* = 1/[σ^2^(*F*_o_^2^) + (0.119*P*)^2^ + 0.0021*P*] where *P* = (*F*_o_^2^ + 2*F*_c_^2^)/3
2363 reflections	(Δ/σ)_max_ < 0.001
174 parameters	Δρ_max_ = 0.75 e Å^−^^3^
0 restraints	Δρ_min_ = −0.23 e Å^−^^3^

### Special details

**Table:**

Geometry. All e.s.d.'s (except the e.s.d. in the dihedral angle between two l.s. planes) are estimated using the full covariance matrix. The cell e.s.d.'s are taken into account individually in the estimation of e.s.d.'s in distances, angles and torsion angles; correlations between e.s.d.'s in cell parameters are only used when they are defined by crystal symmetry. An approximate (isotropic) treatment of cell e.s.d.'s is used for estimating e.s.d.'s involving l.s. planes.
Refinement. Refinement of *F*^2^ against ALL reflections. The weighted *R*-factor *wR* and goodness of fit *S* are based on *F*^2^, conventional *R*-factors *R* are based on *F*, with *F* set to zero for negative *F*^2^. The threshold expression of *F*^2^ > σ(*F*^2^) is used only for calculating *R*-factors(gt) *etc*. and is not relevant to the choice of reflections for refinement. *R*-factors based on *F*^2^ are statistically about twice as large as those based on *F*, and *R*- factors based on ALL data will be even larger.

### Fractional atomic coordinates and isotropic or equivalent isotropic displacement parameters (Å^2^)

**Table:**

	*x*	*y*	*z*	*U*_iso_*/*U*_eq_	
S1	1.45493 (14)	1.26807 (6)	0.93597 (7)	0.0422 (3)	
O1	1.5681 (5)	1.3462 (2)	0.8678 (2)	0.0554 (7)	
O2	1.6472 (5)	1.2080 (2)	1.0081 (2)	0.0634 (7)	
O3	0.3600 (4)	0.95918 (17)	0.60055 (17)	0.0415 (5)	
H3	0.2045	0.9207	0.5865	0.050*	
O4	0.3671 (6)	0.6609 (2)	0.4925 (2)	0.0669 (8)	
O5	0.3457 (5)	0.65735 (18)	0.66724 (18)	0.0505 (6)	
N1	0.8171 (5)	0.8421 (2)	0.5424 (2)	0.0385 (6)	
H1A	0.7266	0.8992	0.5112	0.046*	
H1B	0.8181	0.7816	0.4930	0.046*	
C1	1.2030 (6)	1.1614 (2)	0.8552 (2)	0.0355 (7)	
C2	1.1603 (6)	1.0510 (3)	0.8907 (3)	0.0422 (7)	
H2	1.2735	1.0315	0.9535	0.051*	
C3	0.9497 (6)	0.9707 (3)	0.8325 (2)	0.0421 (7)	
H3A	0.9218	0.8963	0.8561	0.050*	
C4	0.7770 (6)	0.9980 (2)	0.7391 (2)	0.0323 (6)	
C5	0.8256 (6)	1.1086 (2)	0.7039 (3)	0.0397 (7)	
H5	0.7141	1.1282	0.6408	0.048*	
C6	1.0380 (6)	1.1896 (2)	0.7618 (3)	0.0431 (8)	
H6	1.0697	1.2634	0.7377	0.052*	
C7	1.2543 (7)	1.3513 (3)	1.0121 (3)	0.0558 (9)	
H7A	1.1644	1.3006	1.0553	0.084*	
H7B	1.1120	1.3860	0.9643	0.084*	
H7C	1.3767	1.4128	1.0585	0.084*	
C8	0.5434 (6)	0.9065 (2)	0.6795 (2)	0.0348 (7)	
H8	0.4342	0.8766	0.7314	0.042*	
C9	0.6716 (6)	0.8019 (2)	0.6256 (2)	0.0337 (7)	
H9	0.8140	0.7743	0.6814	0.040*	
C10	0.4449 (6)	0.6998 (2)	0.5853 (3)	0.0401 (7)	
C11	0.1378 (8)	0.5547 (3)	0.6424 (3)	0.0554 (9)	
H11A	0.2228	0.4867	0.6148	0.067*	
H11B	−0.0212	0.5704	0.5879	0.067*	
C12	0.0405 (8)	0.5310 (3)	0.7433 (3)	0.0647 (11)	
H12A	0.1986	0.5138	0.7960	0.097*	
H12B	−0.1006	0.4645	0.7289	0.097*	
H12C	−0.0398	0.5994	0.7705	0.097*	

### Atomic displacement parameters (Å^2^)

**Table:**

	*U*^11^	*U*^22^	*U*^33^	*U*^12^	*U*^13^	*U*^23^
S1	0.0315 (4)	0.0384 (5)	0.0528 (5)	−0.0036 (3)	0.0039 (3)	−0.0026 (4)
O1	0.0479 (13)	0.0479 (13)	0.0689 (16)	−0.0132 (10)	0.0156 (12)	0.0027 (12)
O2	0.0454 (13)	0.0540 (14)	0.0788 (18)	−0.0011 (11)	−0.0159 (13)	0.0042 (13)
O3	0.0276 (10)	0.0386 (11)	0.0564 (13)	0.0004 (8)	0.0012 (9)	0.0102 (10)
O4	0.0795 (18)	0.0663 (16)	0.0462 (15)	−0.0263 (13)	0.0091 (13)	−0.0087 (13)
O5	0.0611 (14)	0.0390 (12)	0.0473 (13)	−0.0160 (10)	0.0106 (11)	−0.0009 (10)
N1	0.0321 (12)	0.0403 (14)	0.0422 (15)	0.0027 (10)	0.0053 (11)	0.0040 (11)
C1	0.0334 (14)	0.0299 (15)	0.0425 (17)	0.0007 (12)	0.0090 (13)	−0.0010 (13)
C2	0.0459 (17)	0.0370 (16)	0.0407 (17)	0.0028 (13)	0.0004 (14)	0.0052 (14)
C3	0.0475 (17)	0.0328 (15)	0.0438 (18)	−0.0024 (13)	0.0046 (14)	0.0065 (13)
C4	0.0323 (14)	0.0286 (14)	0.0366 (16)	0.0024 (11)	0.0092 (12)	0.0009 (12)
C5	0.0413 (16)	0.0316 (15)	0.0433 (17)	0.0012 (12)	0.0003 (13)	0.0061 (13)
C6	0.0471 (17)	0.0278 (15)	0.0517 (19)	−0.0006 (13)	0.0023 (15)	0.0072 (14)
C7	0.0475 (18)	0.051 (2)	0.064 (2)	−0.0121 (15)	0.0144 (17)	−0.0194 (17)
C8	0.0319 (14)	0.0327 (15)	0.0395 (17)	0.0013 (12)	0.0051 (12)	0.0069 (13)
C9	0.0323 (14)	0.0304 (14)	0.0368 (16)	0.0024 (12)	0.0027 (12)	0.0031 (12)
C10	0.0412 (16)	0.0309 (15)	0.0464 (19)	0.0025 (13)	0.0057 (14)	0.0008 (14)
C11	0.059 (2)	0.0346 (17)	0.068 (2)	−0.0119 (15)	0.0118 (18)	−0.0042 (16)
C12	0.074 (3)	0.049 (2)	0.074 (3)	−0.0032 (18)	0.021 (2)	0.0179 (19)

### Geometric parameters (Å, °)

**Table:**

S1—O2	1.432 (2)	C4—C5	1.389 (4)
S1—O1	1.436 (2)	C4—C8	1.515 (4)
S1—C7	1.753 (4)	C5—C6	1.378 (4)
S1—C1	1.760 (3)	C5—H5	0.9300
O3—C8	1.412 (3)	C6—H6	0.9300
O3—H3	0.8200	C7—H7A	0.9600
O4—C10	1.198 (4)	C7—H7B	0.9600
O5—C10	1.329 (4)	C7—H7C	0.9600
O5—C11	1.453 (4)	C8—C9	1.544 (4)
N1—C9	1.452 (4)	C8—H8	0.9800
N1—H1A	0.8900	C9—C10	1.518 (4)
N1—H1B	0.8900	C9—H9	0.9800
C1—C6	1.374 (4)	C11—C12	1.475 (5)
C1—C2	1.384 (4)	C11—H11A	0.9700
C2—C3	1.370 (4)	C11—H11B	0.9700
C2—H2	0.9300	C12—H12A	0.9600
C3—C4	1.387 (4)	C12—H12B	0.9600
C3—H3A	0.9300	C12—H12C	0.9600
			
O2—S1—O1	118.80 (15)	H7A—C7—H7B	109.5
O2—S1—C7	108.53 (18)	S1—C7—H7C	109.5
O1—S1—C7	107.05 (17)	H7A—C7—H7C	109.5
O2—S1—C1	108.27 (14)	H7B—C7—H7C	109.5
O1—S1—C1	109.22 (14)	O3—C8—C4	109.8 (2)
C7—S1—C1	103.98 (15)	O3—C8—C9	110.1 (2)
C8—O3—H3	109.5	C4—C8—C9	110.3 (2)
C10—O5—C11	117.5 (3)	O3—C8—H8	108.9
C9—N1—H1A	109.3	C4—C8—H8	108.9
C9—N1—H1B	109.2	C9—C8—H8	108.9
H1A—N1—H1B	109.5	N1—C9—C10	113.8 (2)
C6—C1—C2	120.3 (3)	N1—C9—C8	110.1 (2)
C6—C1—S1	120.6 (2)	C10—C9—C8	110.3 (2)
C2—C1—S1	119.0 (2)	N1—C9—H9	107.4
C3—C2—C1	119.3 (3)	C10—C9—H9	107.4
C3—C2—H2	120.4	C8—C9—H9	107.4
C1—C2—H2	120.4	O4—C10—O5	124.1 (3)
C2—C3—C4	121.4 (3)	O4—C10—C9	125.1 (3)
C2—C3—H3A	119.3	O5—C10—C9	110.8 (2)
C4—C3—H3A	119.3	O5—C11—C12	107.6 (3)
C3—C4—C5	118.4 (3)	O5—C11—H11A	110.2
C3—C4—C8	119.0 (2)	C12—C11—H11A	110.2
C5—C4—C8	122.5 (2)	O5—C11—H11B	110.2
C6—C5—C4	120.4 (3)	C12—C11—H11B	110.2
C6—C5—H5	119.8	H11A—C11—H11B	108.5
C4—C5—H5	119.8	C11—C12—H12A	109.5
C1—C6—C5	120.1 (3)	C11—C12—H12B	109.5
C1—C6—H6	119.9	H12A—C12—H12B	109.5
C5—C6—H6	119.9	C11—C12—H12C	109.5
S1—C7—H7A	109.5	H12A—C12—H12C	109.5
S1—C7—H7B	109.5	H12B—C12—H12C	109.5

### Hydrogen-bond geometry (Å, °)

**Table:**

*D*—H···*A*	*D*—H	H···*A*	*D*···*A*	*D*—H···*A*
O3—H3···N1^i^	0.82	1.97	2.784 (3)	174
N1—H1A···O3^ii^	0.89	2.25	3.088 (3)	158
C7—H7A···O2^i^	0.96	2.57	3.227 (4)	126

Symmetry codes: (i) *x*−1, *y*, *z*; (ii) −*x*+1, −*y*+2, −*z*+1.
